# MicroRNA‐125b in vascular diseases: An updated systematic review of pathogenetic implications and clinical applications

**DOI:** 10.1111/jcmm.14535

**Published:** 2019-07-13

**Authors:** Chia‐Ter Chao, Hsiang‐Yuan Yeh, Tzu‐Hang Yuan, Chih‐Kang Chiang, Huei‐Wen Chen

**Affiliations:** ^1^ Department of Medicine National Taiwan University Hospital BeiHu Branch, National Taiwan University College of Medicine Taipei Taiwan; ^2^ Department of Internal Medicine National Taiwan University College of Medicine Taipei Taiwan; ^3^ Graduate Institute of Toxicology National Taiwan University College of Medicine Taipei Taiwan; ^4^ School of Big Data Management Soochow University Taipei Taiwan

**Keywords:** atherosclerosis, biomarker, endothelial dysfunction, inflammation, microRNA, miR‐125b, neo‐intimal hyperplasia, vascular calcification, vasculopathy

## Abstract

Epigenetic changes, particularly non‐coding RNAs, have been implicated extensively in the pathogenesis of vascular diseases. Specific miRNAs are involved in the differentiation, phenotypic switch, proliferation, apoptosis, cytokine production and matrix deposition of endothelial cells and/or vascular smooth muscle cells. MicroRNA‐125b has been studied in depth for its role in carcinogenesis with a double‐edged role; that is, it can act as an oncogene in some cancer types and as a tumour suppressor gene in others. However, cumulative evidence from the use of advanced miRNA profiling techniques and bioinformatics analysis suggests that miR‐125b can be a potential mediator and useful marker of vascular diseases. Currently, the exact role of miR‐125b in vascular diseases is not known. In this systematic review, we intend to provide an updated compilation of all the recent findings of miR‐125b in vascular diseases, using a systematic approach of retrieving data from all available reports followed by data summarization. MiR‐125b serves as a pathogenic player in multiple vascular pathologies involving endothelia and vascular smooth muscle cells and also serves as a diagnostic marker for vascular diseases. We further provide a computational biologic presentation of the complex network of miR‐125b and its target genes within the scope of vascular diseases.

## 1 BACKGROUND

The impact of cardiovascular disease (CVD) has been increasing in the era of accelerated ageing, multicomorbidity and westernized dietary changes, accompanied by the rising incidence of atherosclerosis, vascular calcification (VC) and other illnesses leading to vascular remodelling. Among CVD, atherosclerosis is the most renowned culprit, significantly contributing to incident ischaemic heart disease (IHD), peripheral vascular disease (PVD) and cerebrovascular disease (CeVD).^1^ Global Burden of Disease Survey already shows that IHD and CeVD are among the top three causes of mortality globally.^2^ Better understanding of the pathogenesis, prompt detection and administration of evidence‐based treatments for vascular diseases remain areas of active research.

Apart from mechanistic insights gleaned from genetic studies of vascular diseases, the role of epigenetic alterations in vascular pathologies is gradually appreciated.^3^ Changes in DNA methylation, chromatin accessibility, histone modifications and expression levels of non‐coding RNAs have all been implicated in the pathogenesis of atherosclerosis.^4^ Atherosclerotic aortas exhibit differentially methylated regions within genes associated with CVD susceptibility.^5^, ^6^, ^7^ Global histone three methylation levels for H3K27me3 reportedly decrease in vascular smooth muscle cells (VSMCs) within atherosclerotic plaques.^8^ In addition, non‐coding RNAs have recently been identified as vital players in the pathogenesis of vascular diseases. Among the family of non‐coding RNAs, microRNAs (miRNAs) are the best characterized members to date; they are short and single‐stranded RNAs with a length between 18 and 25 nucleotides, playing mainly an inhibitory role during gene regulation in assistance with a protein complex termed RNA‐induced silencing complex (RISC).^9^ Each miRNA may have a broad range of targets and may be capable of regulating several physiological processes simultaneously, suggesting that the actions of miRNAs are tissue‐ and species‐specific.^10^

## 2 MICRORNAS AND VASCULAR DISEASES

The pathogenic importance of miRNAs in vascular biology and pathologies cannot be over‐emphasized.^11^, ^12^, ^13^, ^14^ Experiments using mice with a conditional knockout of Dicer in VSMCs demonstrated a retarded cell proliferation and a tendency for neo‐intima formation^15^; vessels with Dicer deletion are thinner and more likely to be dilated with poor contractility than those without.^16^ Dicer‐deficient mice exhibit a global miRNA reduction along with a prominent decline in blood pressure and poor vascular remodelling as well as compromised repair capacity.^17^ Others also demonstrated the influence of specific miRNAs on the differentiation, phenotypic switch, proliferation, apoptosis, cytokine production and matrix deposition of VSMCs. For example, Xie et al^18^ showed that miR‐1 promoted the differentiation of stem cells into VSMCs by suppressing Kruppel‐like factor 4 (KLF4) while inhibited VSMC contractility by down‐regulating SM22 and α‐SMA.^19^ Overexpression of miR‐133a could inhibit the trans‐differentiation of VSMCs into osteoblast‐like cells by decreasing RUNX2 expression, thereby limiting the tendency to develop VC.^20^ Certain miRNAs are pivotal determinants of myogenic vascular tone; animals with defective miR‐143/145 exhibit an increased tendency for developing severe vascular inflammation, adventitial fibrosis and neo‐intimal hyperplasia involving bowel vessels.^21^ In addition, miRNAs also participate extensively in angiogenesis; these miRNAs have been termed angio‐miRs,^22^ although their actions may be context‐dependent.

Traditionally, angio‐miRs include pro‐angio‐miRs (miR‐21, miR‐27b, miR‐31, miR‐126, miR‐130a, miR‐210, miR‐296, miR‐378, etc) and anti‐angio‐miRs (miR‐15, miR‐16, miR‐20a, miR‐20b, miR‐221/222, miR‐320, etc).^23^, ^24^, ^25^, ^26^ These miRNAs act directly or indirectly by modulating vascular endothelial growth factor (VEGF) levels or are stimulated upon VEGF exposure, thus either promoting or inhibiting angiogenesis. However, subsequent studies uncover a broader range of miRNAs participating in the pathogenesis of vascular diseases, constituting vascular miRs. Examples of vascular miRs include miR‐143, miR‐145, miR‐221, miR‐322 and miR‐424, whose overexpression in VSMCs is associated with phenotypic switch, altered migratory and proliferative ability, and neo‐intimal formation.^27^, ^28^, ^29^, ^30^, ^31^ MiR‐125b is a relative newcomer in the spectrum of vascular miRs.

## 3 MIR‐125B: AN INTRODUCTION

MiR‐125b is the human orthologue of the miRNA *lin‐4*, which is among the first few miRNAs discovered in *Caenorhabditis elegans*.^32^ In human cells, miR‐125b is ubiquitously expressed, with particularly high levels in tissues such as brain, ovaries, thyroid glands and pituitary gland. Human miR‐125b belongs to the miR‐125b family, which includes miR‐125a (on chromosome 19q13), miR‐125b‐1 (on chromosome 11q24) and miR‐125b‐2 (on chromosome 21q21); miR‐125b‐1 and miR‐125b‐2 produce identical products.

MiR‐125b exhibits a Janus‐faced influence in several pathophysiological processes, especially during the course of cellular oncogenic transformation.^33^ MiR‐125b loss of function has been observed in several tumour types including non–small‐cell lung cancer, breast, ovary, cervical, head and neck cancer, glioma and melanoma,^34^, ^35^, ^36^, ^37^ while promotion of miR‐125b signalling has been reported in studies on gastric, colon, pancreatic cancer and leukaemia.^38^, ^39^, ^40^ Some of these changes in transformed cells are related to a passive stance for miR‐125b in cellular transformation because of the hypermethylation in its promoter region^41^ and/or chromosomal translocation leading to its up‐regulation.^42^ However, cumulative evidence suggests that miR‐125b exhibits substantial influences on cellular proliferation, apoptosis or survival, and lineage‐specific differentiation, by targeting p53, HER2/3, Bcl, Bak and PI_3_K/Akt pathways.^43^, ^44^, ^45^ These effects in non‐vascular tissues underline and summarize the mechanistic importance of miR‐125b known previously.

## 4 MICRORNA‐125B IN VASCULAR DISEASES

MiR‐125b was first implicated in vascular pathogenesis when researchers examined miRNA microarray results using VSMCs of diabetic *db/db* mice and *db*/+ littermates.^46^ MiR‐125b was significantly up‐regulated in the former and increased the expression of interleukin‐6 (IL‐6) and monocyte chemoattractant protein‐1 (MCP‐1). Since then, cumulative evidence suggests that miR‐125b participates extensively in the pathogenesis and clinical diagnosis of vascular diseases. In the following sections, we will use a systematic approach through retrieving data from all available reports in the existing literature, followed by data summarization, to illustrate the under‐recognized importance of miR‐125b in vascular diseases.

**Figure 1:**
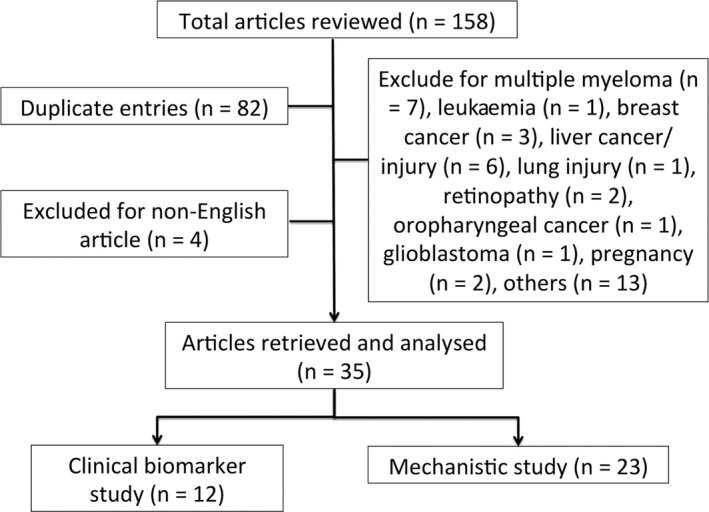
The algorithm of retrieval of studies from the literature and application of selection criteria

We systematically searched through evidence sources including PubMed, MEDLINE, and EMBASE databases with results up to February 28th, 2019, using the keywords “microRNA‐125b” or “miR‐125b”, and those related to vascular diseases (“vasculopathy”, “vascular”, “atherosclerosis”, “vasculitis”, “thrombosis”, “vessel”, “artery”, “vein”, “endothelium”, or “vascular smooth muscle cell”) (Figure 1). The inclusion criteria included both human and animal studies addressing miR‐125b and vascular diseases, regardless of whether the miR‐125b was the primary focus of study or not. The exclusion criteria consisted of duplicated or non‐English articles, database reports, and reports that addressed cancer or non‐vascular diseases, such as lung and liver injury, and pregnancy. The abstract of each report was independently reviewed by two authors (CTC and HYY) in a blind manner, with controversies resolved by a consensus meeting with all co‐authors.

**Figure 2:**
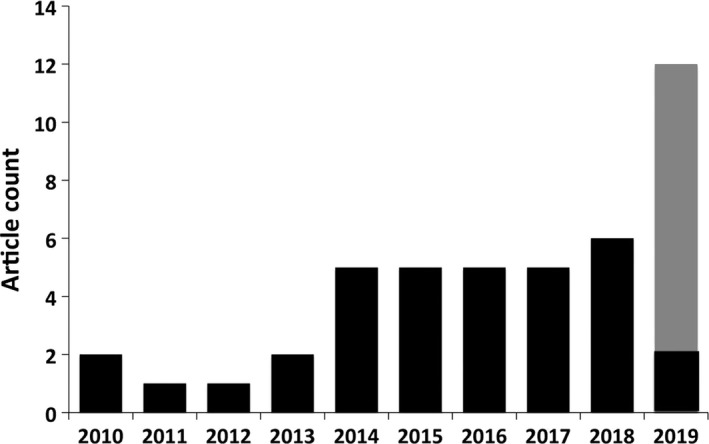
A bar chart illustrating the temporal trend of the number of published articles addressing miR‐125b and vascular diseases. Black bar represents published article count. Grey bar represents projected article count in 2019

A total of 158 published articles were retrieved during the study period. After applying the exclusion criteria, 35 articles were finally included with full text reviewed (Figure 1).^46^, ^47^, ^48^, ^49^, ^50^, ^51^, ^52^, ^53^, ^54^, ^55^, ^56^, ^57^, ^58^, ^59^, ^60^, ^61^, ^62^, ^63^, ^64^, ^65^, ^66^, ^67^, ^68^, ^69^, ^70^, ^71^, ^72^, ^73^, ^74^, ^75^, ^76^, ^77^, ^78^, ^79^, ^80^ If aligned based on the date of publication, it was observed that the number of studies addressing miR‐125b and vascular diseases increases gradually over time since the first report that was published in 2010 (Figure 2). For the clarity of presentation, we divided the content into two parts: the first part discussing miR‐125b as a pathogenic player in different types of vascular diseases and the second part depicting miR‐125b as a clinical marker for these diseases.

## 5 MICRORNA‐125B: A PATHOGENIC ROLE IN VASCULAR DISEASES

### 5.1 Genetic background involving miR‐125b and the risk of vascular events

It has long been known that genetic traits, or sequence polymorphisms, are important risk factors for vascular events across populations,^81^ and results from association studies between single nucleotide polymorphism (SNP) and phenotypes often provide useful information regarding the regulatory spectrum of such genes. Several prior studies have shown that sequence polymorphisms of binding sites of miR‐125b and its targets, including bone morphogenetic protein receptor^82^ and TP53,^83^ influence the risk of carcinogenesis and ophthalmologic disorders. Such a relationship between miR‐125b and the risk of vascular diseases had not been described, until recently. By integrating results from genome‐wide miRNA expression quantitative trait locus mapping, SNPs annotated by the 1000 Genomes Project, and phenotypes from prior miRNA genome‐wide association studies, researchers have revealed that polymorphisms of *cis*‐acting regulatory elements for miR‐125b were highly correlated with total cholesterol and high‐density lipoprotein cholesterol levels among participants.^84^ This finding, although still preliminary, underlines the possibility that miR‐125b may modulate certain risk factors for vascular diseases, especially atherosclerosis.

### 5.2 Vascular effect: miR‐125b in endothelial cells (ECs)

A large proportion of the retrieved studies have focused on the influence of miR‐125b on ECs. As described above, miR‐125b is not a conventional angio‐miR, and prior data based on miRNA profiling from ECs and VSMCs failed to uncover miR‐125b as a differentially expressed miRNA.^85^, ^86^ Li et al^47^ first reported that mice ECs abundantly expressed miR‐125b compared to fibroblasts or immunocytes, and miR‐125b expression in aortas was higher than that in skeletal muscles, heart, liver and kidneys. They further showed that oxidized low‐density lipoprotein (ox‐LDL), a key player of atherosclerosis, suppressed EC miR‐125b expression and promoted endothelin‐1 synthesis, while miR‐125b expression restoration could attenuate endothelin‐1 expression. Animal experiments also affirmed that spontaneously hypertensive rats exhibited significantly down‐regulated aortic miR‐125b and up‐regulated endothelin‐1 expression,^47^ suggesting that miR‐125b could influence the vascular tone. Using a lower limb vaso‐occlusion model, Muramatsu et al^50^, ^87^ showed that baseline miR‐125b expression in ECs was not high but was inducible upon ischaemic and pro‐angiogenic stimuli exposure; up‐regulation of miR‐125b in ECs could suppress VE‐cadherin mRNA translation, reduce endothelial permeability/leakage, and modulate the risk of subsequent atherosclerosis. Similarly, Zhou et al^57^ showed that in heat‐injured human umbilical vascular ECs (HUVECs), miR‐125b up‐regulation was associated with decreased expression of human epidermal receptor 2 (HER2) and VEGF; neo‐angiogenesis and vascularization have been shown to play a vital role in plaque growth and destabilization, and anti‐angiogenetic therapies have been shown to ameliorate the progression of atherosclerosis.^88^ In in vitro atherosclerosis models, such as ox‐LDL‐treated HUVECs, miR‐125b was found to be suppressed over time, accompanied by the up‐regulation of podocalyxin. Forced up‐regulation of miR‐125b inhibited endothelial proliferation and migration, reducing vascular endothelial‐cadherin (VE‐cadherin) and intercellular adhesion molecule‐1 (ICAM‐1) expression, and thus, alleviating the adverse effects of atherogenesis.^59^

However, miR‐125b may exhibit unexpected adverse effects over other pathogenic phenotypes during vascular disease progression. In vitro experiments revealed that miR‐125b expression levels increased progressively with senescence of ECs, accompanied by up‐regulation of inflammatory markers, such as MCP‐1.^49^ Zhong et al^58^ further demonstrated that high glucose exposure induced a progressive surge in miR‐125b expression over time, which correlated with a persistent up‐regulation of nuclear factor (NF‐κB), IL‐6 and MCP‐1 in human aortic ECs by directly targeting tumor necrosis factor alpha‐induced protein 3 (TNFAIP3) in vitro. Endothelial inflammation is an important predecessor of endothelial dysfunction, which is subsequently followed by atherosclerosis or loss of vascular compliance.

**Figure 3:**
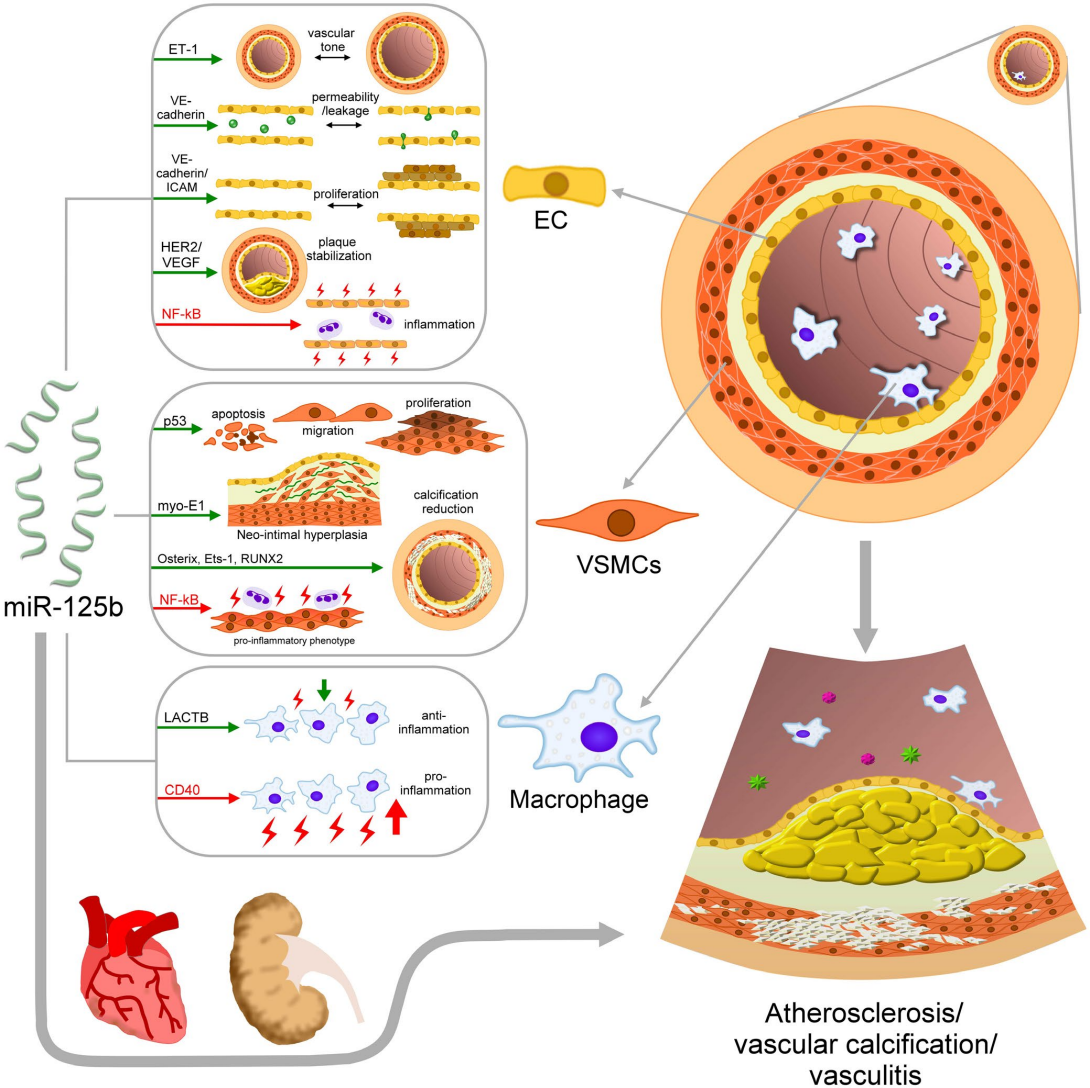
A schematic diagram showing the known mechanisms mediating the dual‐faced roles of miR‐125b in the pathogenesis of vascular diseases. Direct effects of miR‐125b on vascular diseases can be exemplified by its influences on ECs, VSMCs and macrophages, while indirect effects are shown in the lower part, through other organ crosstalk. Green arrows indicate vasculo‐protective effect of miR‐125b, while red arrows suggest vasculopathic effect. EC, endothelial cells; ET‐1, endothelin‐1; VEGF, vascular endothelial growth factor; VSMC, vascular smooth muscle cells

Taken together, miR‐125b exhibits significant effects on various phenotypes of ECs during vascular diseases, which could be double‐edged, similar to those observed during oncology studies involving miR‐125b. An increase in miR‐125b favourably affects vasomotor tone, reduces vascular permeability and affects endothelial proliferation/migration; most of the miR‐125b actions are anti‐atherogenic in nature. However, miR‐125b confers a pro‐inflammatory phenotype to ECs, which is associated with a higher risk of vascular diseases (Figure 3). Before we can reach a definitive conclusion, it should be noted that the nature of ECs on which studies were carried out (human vs animals, embryonic vs adult, aorta vs peripheral vessels, etc) is different across different studies, and results of some of these studies have not been validated in vivo or in human tissues/samples.

### 5.3 Vascular effect: miR‐125b in vascular smooth muscle cells

Earlier studies showed that the expression level of miR‐125b decreased significantly in injured rat carotid artery, and the decrease persisted for 7 days,^89^ but whether this change was related to ECs, VSMCs or other supporting cells remained unclear. The importance of miR‐125b was not recognized until a decade ago, when Villeneuve et al^46^ showed that miR‐125b was significantly up‐regulated in aortic VSMCs of diabetic rats compared to non‐diabetic littermates, and treatment with miR‐125b mimics enhanced the expression of IL‐6 and MCP‐1 in VSMCs by regulating histone modifications. However, different findings were observed in later studies, indicating the protective role of miR‐125b in vascular pathologies involving VSMCs. For example, in chronic kidney disease (CKD), Chen et al^51^ reported that both sera and aortic miR‐125b expression decreased substantially in rats with CKD. In VSMCs from atherosclerotic mice, Cao et al^66^ observed that miR‐125b levels decreased after homocysteine‐induced VSMC proliferation, and miR‐125b silenced p53 via DNA methylation by targeting DNMT3b in vitro and in vivo. MiR‐125b has been shown to suppress VSMC proliferation and migration, and mice with experimental carotid artery injury exhibited less neo‐intimal formation and a lower degree of injury after miR‐125b transfection.^77^ A more recent study elucidates the role of exosome‐mediated transfer of miR‐125b in vascular disease; Wang et al^80^ showed that VSMCs might internalize extracellular vesicles containing miR‐125b, which suppressed myosin 1E expression in these cells and ameliorated the severity of intimal hyperplasia.

The role of miR‐125b in mediating vascular diseases other than atherosclerosis has been gradually recognized. Goettsch and colleagues reported that osteogenic trans‐differentiation of human coronary VSMCs was accompanied by reduced expression of miR‐125b, which was confirmed in calcified aortas of ApoE knockout mice.^48^ They further identified that osterix may be a direct target of miR‐125b in this process. In an in vitro model of VSMCs obtained from CKD rats, Chen et al^51^ reported that miR‐125b expression decreased significantly compared to the VSMCs obtained from normal rats, whereas RUNX2, an osteoblast marker, was up‐regulated along with aortic calcification. This is further corroborated in another study using primary rat VSMCs exposed to osteogenic media, which showed that miR‐125b decreased with VSMC calcification and that restoration of miR‐125b in these cells could suppress the process of calcification by suppressing Ets‐1.^55^ We have previously focused on the influence of miR‐125b on VC in vitro and in vivo using both rat and human VSMCs and observed that VSMCs treated with miR‐125b mimics exhibited a lower tendency for phosphate‐induced calcification compared to that by controls. In addition, CKD rats with a higher severity of VC exhibited attenuated aortic miR‐125b expression compared to non‐CKD animals or those with less severe VC.^70^ Further, we have demonstrated that miR‐125b overexpression suppressed osteoblast markers including RUNX2 and osteocalcin in VSMCs. Based on the above findings, the consistency in the effect of miR‐125b on the phenotype of VC indicates a potentially important therapeutic role of miR‐125b in VC. Nonetheless, a prior report using osteoprotegerin‐knockout mice with coronary VC has shown that VSMC miR‐125b levels do not differ between experimental and control groups,^71^ which indicates that the genetic background of animal models may influence the effect of miR‐125b on VC.

To further explore the molecular linkage of miR‐125b and calcification‐related pathways, we had conducted a bioinformatics linkage analysis with validation in clinical samples of patients with uraemic VC.^79^ We found that the levels of key molecules involved in mineral bone disease, including osteoprotegerin, fetuin‐A and fibroblast growth factor‐23, were significantly correlated with the serum levels of miR‐125b in uraemic patients, and through direct and indirect regulation of these molecules, miR‐125 was capable of altering the risk of VC in patients with a higher risk of developing such vascular complications.

### 5.4 Pathogenesis: miR‐125b and miscellaneous cells/tissues in vascular diseases

Apart from ECs and VSMCs, other types of cells, including resident macrophages, are key players in vascular diseases as well. Studies in THP‐1 macrophages have suggested that miR‐125b directly targets β‐lactamase mRNA by binding to its 3′‐untranslated region and reduces MCP‐1 secretion in these cells.^64^ On the contrary, another report on ApoE knockout mice showed that miR‐125b levels were higher in atherosclerotic plaques and lipopolysaccharide‐stimulated macrophages; in these mice, miR‐125b was up‐regulated by CD40 signalling.^65^ This dual effect of miR‐125b in macrophages dwelling in atheroma is reminiscent of that in different cancer cells and may arise from the differences in the cell lines being tested, differences in the stimuli on macrophages, and the comparators of miR‐125b levels regarding tissue‐level validation. Nonetheless, these findings confirmed that the effects of miR‐125b can be mediated in the macrophages during the pathogenesis of atherosclerosis.

MiR‐125b has also been found to participate in the process of valvular calcification and the recovery and repair of renal, cerebrovascular and myocardial injury, most of which modulate hemodynamic status and indirectly cause vascular injuries in its aftermath. Ohukainen et al^61^ found miR‐125b was up‐regulated in cultured human THP‐1 macrophages, and through indirectly enhancing C‐C motif chemokine ligand‐4(CCL4) expression, miR‐125b might participate in the pathogenesis of aortic valve calcification. Wang et al^56^ in a mouse model of myocardial ischaemia/reperfusion injury reported that miR‐125b up‐regulation could ameliorate the extent and severity of myocardial infarction through attenuating the activation of NF‐κB and myocardial p53 and Bak1 expression. Using a coronary artery ligation model, another group of researchers showed that miR‐125b was stress‐inducible, suppressed cardiomyocyte apoptosis and promoted survival through inhibiting Bak1 expression and activating phosphor‐AKT signalling.^72^ In this sense, miR‐125b has been cited as being cardio‐protective by some groups.^73^

In addition, a rise in tissue miR‐125b levels has been observed in a model of renal ischaemia reperfusion and was reversible upon dedicated treatment against renal damage,^68^ whereas a similar finding has been reported in a model of cerebral ischaemia reperfusion injury.^75^ This beneficial effect has been attributed to an altered redox balance in the involved tissues, although whether this mechanism is applicable to other tissues remains unclear.

## 6 SUMMARY

**Figure 4:**
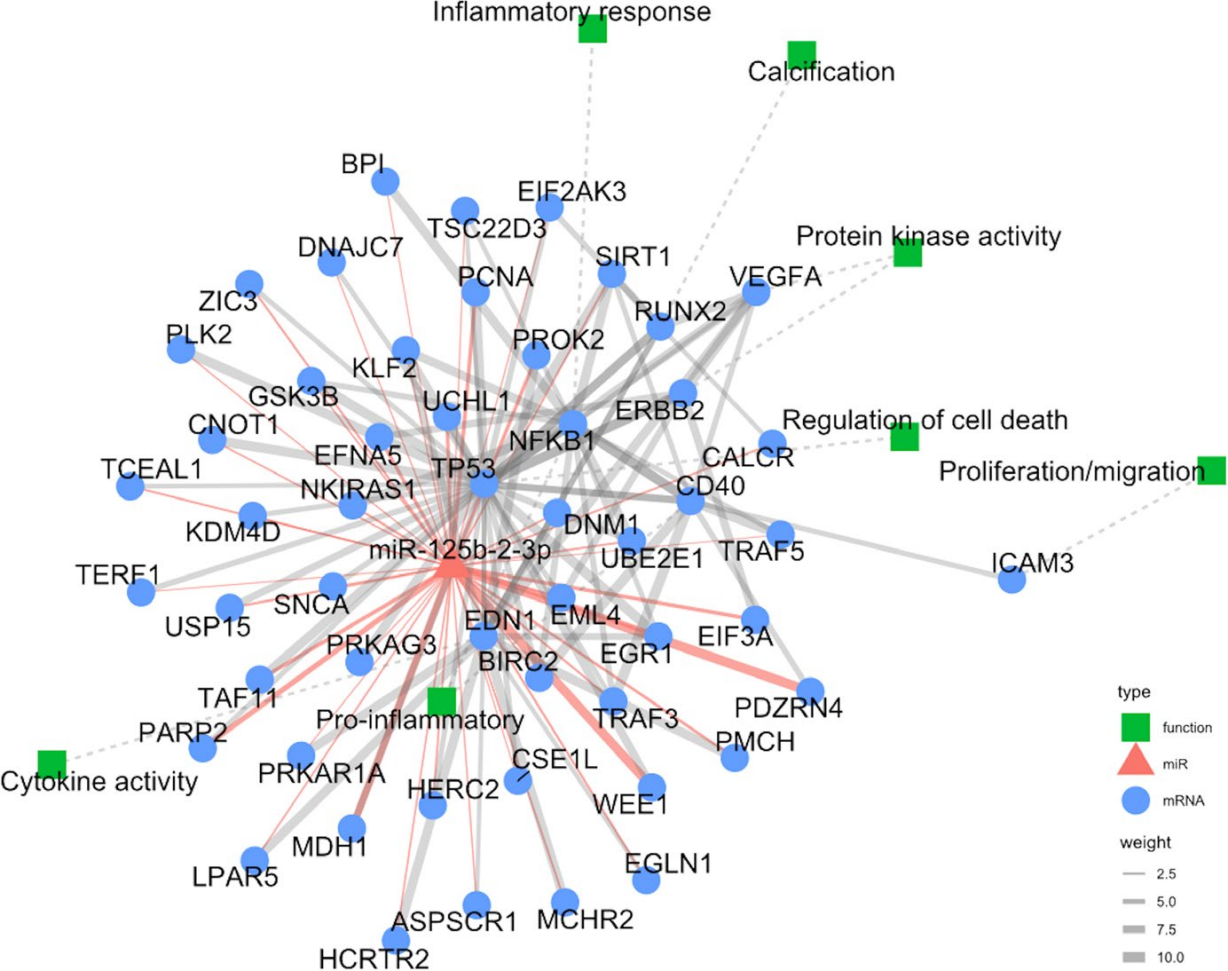
A global view of the associated functions and pathways of miR‐125b. The straight arrow between miR‐125b (triangle) and its target genes (circle) has been validated by experimental data or literature, and the dashed arrow denotes the associated functions (rectangle) regulated by each mRNA. The width of edge is mapped to its strength based on the aggregated prediction score. The interactome presents the mechanism of action of miRNA‐125b involved in a set of interacting molecular network. miR, microRNA

Based on findings from the recent literature, it is evident that the vascular effects of miR‐125b can be regarded as both adverse and favourable, depending on the culprit cells and the types of vascular pathology (Figure 3). Through the regulation of multiple downstream targets according to existing studies, miR‐125b can modulate the process of vascular diseases ranging from atherosclerosis, vascular inflammation to VC (Table 1). We further used the computational biology approach to uncover potential biological processes affected by miR‐125b, based on the molecular targets summarized in Table 1. We collected the predicted and validated miRNA–target interactions from the most commonly cited prediction algorithms to perform target enrichment analysis. Target genes of miRNA‐125b occur in at least two sources were identified, with hypergeometric tests used to estimate the significance of enrichment, followed by further extension and target analysis using the latest available version of STRING database (https://string-db.org/). Only first‐level interactions between target mRNAs and their neighbours were detected. Function and pathway enrichment analyses were undertaken using DAVID and GSEA toolkits, and we mapped the target mRNAs of miRNA‐125b to the parental protein‐protein interaction network (Figure 4). The close connections between miR‐125b and its target genes as well as related biologic processes exemplify the potential role of miR‐125b in the pathogenesis of vascular diseases. The interplay between these molecules likely shapes the true influence of miR‐125b in various vascular pathologies.

**Table 1:** Summary of experimental studies investigating the regulators and effectors of miR‐125b in vascular diseases

Experimental setting	Vascular phenotypes	Regulators	Targets	Reference
In vitro + In vivo	Hypertension	Oxidized LDL	Endothelin‐1	47
In vitro	Vascular inflammation	–	MCP‐1, IL‐6, Suv39h1	46
In vitro	Vascular calcification	–	Osterix, SP7	48
In vitro	Angiogenesis	VEGF	VE‐cadherin	50
In vitro	Vascular calcification	–	Ets‐1	55
In vitro	Vascular inflammation	–	MCP‐1, IL‐6, p65	58
In vitro	Atherosclerosis	–	Podocalyxin	59
In vivo	Valvular calcification	–	CCL4	61
In vitro	Atherosclerosis	–	LACTB	64
In vivo	Atherosclerosis	CD40, NF‐κB	–	65
In vitro	Atherosclerosis	–	DNMT3b	66
Ex vivo + In vivo	Peripheral vascular disease	‐	SRF	77

Abbreviations: IL‐6, interleukin‐6; LDL, low‐density lipoprotein; MCP, monocyte chemoattractant protein; SRF, serum response factor; VEGF, vascular endothelial growth factor.

## 7 MICRORNA‐125B: A POTENTIAL BIOMARKER FOR VASCULAR DISEASES

miRNAs exhibit tissue‐specific and time‐dependent roles in physiological and pathological processes. As small RNAs are stable in biological fluids and remain detectable even after prolonged storage, increasing numbers of studies are being devoted to the utilization of circulating miRNAs as biomarkers to evaluate the risks of incidence or progression of a disease. Existing studies have revealed the mechanism of packaging of miRNAs into extracellular vesicles, including but not limited to exosomes and their shuttling to neighbouring or even distant tissues. The identification of these mechanisms greatly facilitates the diagnostic importance of miRNAs in multiple disorders, particularly in vascular diseases.^90^

MiR‐125b is no exception in this perspective. During our systematic literature search, we identified 12 articles examining the diagnostic utility of miR‐125b in different types of cardiovascular diseases, with the first report published 5 years ago (Table 2).^52^, ^53^, ^54^, ^60^, ^62^, ^63^, ^67^, ^69^, ^70^, ^74^, ^76^, ^78^ Among these diseases, we found that coronary artery disease/coronary events (n = 4) and acute stroke (n = 3) are the most common diseases in which the role of circulating miR‐125b has been investigated, followed by intracranial aneurysms (n = 2), abdominal aortic aneurysm (n = 1), heart failure (n = 1) and VC (n = 1) (Table 2). The similarities and differences in these reports are briefly discussed below.

**Table 2:** Clinical studies investigating miR‐125b in patients with vascular diseases

Articles	Sample size	Origin	Categorization	Disease type	Relationship	Reference
Huang et al. (2014)	676	Plasma	Low vs High miR‐125b	Acute myocardial infarction	OR 4.27 for disease (2.84‐6.41)	52
Liu et al. (2014)	12	Tissue	Disease vs control samples	Intracranial aneurysm	FC 0.29 (*P* = .04)	53
Sepramaniam et al. (2014)	287	Whole blood	Disease vs control samples	Acute stroke	FC 1.795 (*P* = .007)	54
Ding et al. (2015)	124	Plasma	Disease vs control samples	Coronary heart disease	FC 0.778 (*P* = .055)	60
Marques et al. (2016)	17	Plasma (coronary sinus)	Disease vs control samples	Congestive heart failure	FC 0.27 (*P* = .023)	62
Jia et al. (2016)	309	Plasma	Disease vs control samples	Acute coronary syndrome	FC 4.46 (*P* = .008)	63
Bo et al. (2017)	61	Whole blood	Disease vs control samples	Intracranial aneurysm	Predicted to be altered	67
Tiedt et al. (2017)	380	Plasma	Disease vs control samples	Acute ischaemic stroke	FC 2.54 (*P* < .0001)	69
Chao et al. (2017)	223	Serum	Low vs High (/control)	Vascular calcification	OR 1.41 for disease (*P* = .03) HR 7.14 for progression (*P* < .01)	70
Courtois et al. (2018)	57	Plasma	Disease vs control samples	Abdominal aortic aneurysm	Lower (*P* < .05)	74
Gui et al. (2018)	205	Serum	Disease vs control samples	Acute ischaemic stroke	FC 1.372 (*P* = .019)	76
Kay et al. (2019)	121	Plasma	Disease vs control samples	Coronary or carotid atherosclerosis	Lower	78

Abbreviations: FC, fold change; HR, hazard ratio; OR, odds ratio.

Huang and colleagues used a mass parallel sequencing of plasma extracted from 20 patients with myocardial infarction and 20 individuals without myocardial infarction and identified that the circulating miR‐125b level was significantly low in myocardial infarction patients.^52^ Their findings were validated in two independent cohorts with more than 150 patients in each group, and the addition of circulating miR‐125 levels into diagnostic criteria further outperformed the existing risk factors. Ding et al^60^ derived similar findings by showing that patients with coronary heart disease had significantly lower plasma miR‐125b levels compared to those without coronary heart disease, although the differences were not statistically significant. Additionally, a recent study reported that plasma miR‐125b levels were lower among lupus patients with coronary or carotid atherosclerosis than in lupus patients without coronary or carotid atherosclerosis; the data were confirmed in an independent cohort study.^78^ Importantly, the degree of estimated differences in risk between disease and control groups is similar across various studies (Table 2), lending support to the diagnostic utility of miR‐125 in atherosclerotic vascular diseases. However, in a smaller cohort, Jia et al^63^ discovered that patients with acute coronary syndrome exhibited significantly higher plasma levels of miR‐125b than those without acute coronary syndrome, unlike previous reports. This may relate to the sample size, patient groups being studied (the former vs the latter, myocardial infarction only or stable coronary atherosclerosis vs acute coronary syndrome), timing of blood tests, and potential differences in risk factor profiles.

Acute stroke represents the second most crucial diagnostic application of circulating miR‐125b (Table 2). Sepramaniam et al^54^ first characterized the differences in expression of miRNAs between patients with and without acute stroke using miRNA profiling and found that miR‐125b levels from whole blood were significantly higher among those with acute stroke compared to patients without acute stroke. This change in blood and brain levels of miR‐125b was confirmed in a parallel rat stroke model in the same study. A subsequent study, which enrolled more patients, revealed that plasma miR‐125b levels rose acutely after the development of acute ischaemic stroke and was restored 2 days later compared to non‐stroke patients.^69^ Gui et al^76^ further demonstrated that cardioembolic stroke patients exhibited significantly higher circulating miR‐125b levels than healthy controls, while patients with stroke because of other origins did not exhibit such discrepancy. Their findings raised the possibility that miR‐125b levels may vary depending upon the type of acute ischaemic stroke, although an increased circulating miR‐125b level is typical among acute stroke patients.

Aneurysms of different anatomical sites represent another area in which miR‐125b has been used as a diagnostic tool. Liu et al^53^ conducted an miRNA profiling of samples from intracranial aneurysms and found that miR‐125b expression was significantly higher in samples from intracranial aneurysms than in those from normal intracranial arteries. Another bioinformatics analysis predicted that blood miR‐125b levels are altered significantly in patients with ruptured intracranial aneurysms, although such changes were not confirmed in clinical specimens.^67^ However, for abdominal aortic aneurysms, Curtois et al^74^ demonstrated that those with rupture‐prone abdominal aneurysms exhibited significantly lower plasma miR‐125b levels compared to those without such aneurysms. Such differences between aneurysms of different sites may result from the diversity in the aetiology or structures of aneurysms and the ethnicity of patients being tested. More studies are needed in this respect for reaching a definitive conclusion.

Vascular calcification is a relatively new vascular pathology phenotype for which circulating miR‐125b can be of diagnostic value. We used a translational platform, incorporating an in vitro VSMC model, in vivo animal VC model and sera from patients with uraemic VC to explore whether miR‐125b could be a valid biomarker for diagnosing VC.^70^ We revealed that both tissue and sera miR‐125b levels were significantly lower among animals with more severe VC. These findings were validated in two independent cohorts of uraemic patients with VC. Finally, low serum miR‐125b level could be a predictive factor of VC progression after patient follow‐up, further stressing the importance and utility of miR‐125b as a biomarker. Consequently, it is likely that miR‐125b could become clinically useful in the future for the diagnosis of multiple cardiovascular diseases, ranging from atherosclerosis, VC, coronary and CeVD, to myocardial infarction, stroke and aneurysms, although the direction of change will depend on the disease being studied.

## 8 CONCLUSIONS

Unlike classical angio‐miRs, which were named after their role in angiogenesis was elucidated more than a decade ago, the role of miR‐125b was initially recognized in the field of oncology, with its dual‐faced role of an oncogene in certain cancers and of a tumour suppressor in others. MiR‐125b, a vascular miR, is now known to primarily participate in the pathogenesis of atherosclerosis, VC and vascular wall inflammation, whereas its applicability as a diagnostic marker in cardiovascular diseases including coronary atherosclerosis, myocardial infarction, heart failure, stroke, aneurysm formation and VC is gradually being unfolded. It has been speculated that miR‐125b could be a promising diagnostic marker and a potential therapeutic tool in the armamentarium against several vascular diseases in the future.

## CONFLICT OF INTEREST

The authors have no relevant financial or non‐financial competing interests to declare in relation to this manuscript.

## AUTHOR CONTRIBUTION

CTC, CKC and HWC designed the study; CTC, HYY and HWC involved in literature survey and analysis; and CTC, HYY, THY, CKC and HWC drafted the manuscript. All authors approved the final version of the manuscript.

## SPONSOR'S ROLE

The sponsors have no role in the study design, data collection, analysis and result interpretation of this study.

## Data Availability

Data will be available upon reasonable request.
